# High glucose microenvironment accelerates tumor growth via SREBP1-autophagy axis in pancreatic cancer

**DOI:** 10.1186/s13046-019-1288-7

**Published:** 2019-07-11

**Authors:** Cancan Zhou, Weikun Qian, Jie Li, Jiguang Ma, Xin Chen, Zhengdong Jiang, Liang Cheng, Wanxing Duan, Zheng Wang, Zheng Wu, Qingyong Ma, Xuqi Li

**Affiliations:** 1 Department of Hepatobiliary Surgery, The First Affiliated Hospital of Xi’an Jiaotong University, Xi’an, 710061 China; 2 Department of Anesthesiology, The First Affiliated Hospital of Xi’an Jiaotong University, Xi’an, 710061 China; 3 Department of General Surgery, The First Affiliated Hospital of Xi’an Jiaotong University, Xi’an, 710061 China

**Keywords:** Pancreatic cancer, High glucose, SREBP1, Autophagy

## Abstract

**Background:**

Diabetes is recognized to be a risk factor of pancreatic cancer, but the mechanism has not been fully elucidated. Sterol regulatory element binding protein 1 (SREBP1) is an important transcription factor involved in both lipid metabolism and tumor progression. However, the relationship between high glucose microenvironment, SREBP1 and pancreatic cancer remains to be explored.

**Methods:**

Clinical data and surgical specimens were collected. Pancreatic cancer cell lines BxPc-3 and MiaPaCa-2 were cultured in specified medium. Immunohistochemistry (IHC) and western blotting were performed to detect the expression of SREBP1. MTT and colony formation assays were applied to investigate cell proliferation. Immunofluorescence, mRFP-GFP adenoviral vector and transmission electron microscopy were performed to evaluate autophagy. We used streptozotocin (STZ) to establish a high glucose mouse model for the in vivo study.

**Results:**

We found that high blood glucose levels were associated with poor prognosis in pancreatic cancer patients. SREBP1 was overexpressed in both pancreatic cancer tissues and pancreatic cancer cell lines. High glucose microenvironment promoted tumor proliferation, suppressed apoptosis and inhibited autophagy level by enhancing SREBP1 expression. In addition, activation of autophagy accelerated SREBP1 expression and suppressed apoptosis. Moreover, high glucose promotes tumor growth in vivo by enhancing SREBP1 expression.

**Conclusion:**

Our results indicate that SREBP1-autophagy axis plays a crucial role in tumor progression induced by high glucose microenvironment. SREBP1 may represent a novel target for pancreatic cancer prevention and treatment.

## Background

Pancreatic cancer (PC) is one of the deadliest types of cancer, it is highly malignant and has a low rate of diagnosis in early stage [1, 2]. Based on the latest data from the American Cancer Society, PC ranks among the top ten cancers in the number of estimated new cases as well as the estimated number of deaths. Additionally, the 5-year survival rate of PC is the lowest of all 15 types of cancer at only 9% [3]. Moreover, there have been no breakthroughs in the early detection of PC. Currently, many risk factors are thought to be closely related to PC tumorigenesis, such as smoking, alcohol consumption, chronic pancreatitis, obesity and diabetes mellitus (DM) [2, 4–8].

According to previous studies, nearly one-third of PC patients have DM, and there is a greater chance for DM patients to develop PC [9–11]. Our previous studies demonstrated that hyperglycemia accelerates PC progression by increasing reactive oxygen species (ROS) levels [12], promoting epithelial-mesenchymal transition (EMT) [13], and aggravating hypoxia [14]. In addition, we demonstrated that hyperglycemia promotes perineural invasion both in vitro and in vivo [15]. Nonetheless, much remains to be learned about the tumorigenesis and tumor progression mechanisms mediated by high glucose microenvironment.

In general, cancer cells require a considerable nutrient supply for their rapid growth, and glucose has been proven to be their main source of energy [16]. Due to its extensive desmoplastic reaction, PC is characterized by hypovascularization [17], and pancreatic cancer cells are accordingly more dependent on a high level of glycolysis rather than oxidative phosphorylation [17–19]. Autophagy is a cellular process that is closely related to energy supply [20]. Basal autophagy, which is essential for maintaining homeostasis, provides energy for cells by degrading proteins and organelles. Overall, autophagy is activated to maintain cell survival when the energy supply is insufficient [21]. In PC, autophagy is thought to be a protective factor during tumorigenesis [21] as well as a factor contributing to proliferation [22].

Sterol regulatory element-binding protein 1 (SREBP1) is a transcription factor that is of vital importance in lipid metabolism [23, 24]. Considering the basic function of SREBP1, there have been some predictions regarding its role in type 2 diabetes, cancer, immune system, autophagy and neuroprotection [25], and the role of SREPB1 in tumor progression has recently been explored. SREBP1 is overexpressed in several tumor types, and it has been confirmed to facilitate tumor proliferation and invasion [26–30].

Here, we examine correlations among high glucose microenvironment, autophagy and SREBP1 to elucidate the possible mechanism underlying the relationship between diabetes and PC. We hypothesize that high glucose microenvironment regulates autophagy levels in PC by upregulating SREBP1 and thereby promoting tumor progression. The findings reveal a potential novel target in preventing tumor progression.

## Methods

### Collection of human tissue and clinical data

The human PC and normal pancreas specimens used in this study were obtained from the Department of Hepatobiliary Surgery, the First Affiliated Hospital of Xi’an Jiaotong University. All patients signed informed consent for sample collection. Histological analyses were performed as previously described [31]. We collected clinical data for 267 patients diagnosed with PC from Jan 2015 to Sep 2016 who were treated at the First Affiliated Hospital of Xi’an Jiaotong University. We chose 110 patients who underwent surgery and had a definitive postoperative pathological diagnosis of PC for further study. We then collected their clinical data, including basic information, fasting blood glucose level before surgery and the other data listed in Table 1. Patients were divided into two groups according to their preoperative blood glucose level: normal glucose group, below 6.1 mmol/L and high glucose group, over 6.1 mmol/L. From Jan 2016 to Mar 2017, we followed all 110 patients via phone calls or outpatient visits. The mean follow-up time was 4.6 ± 3.8 months.

**Table 1:** Correlation between blood glucose level and the clinicopathologic characteristics of PC patients

Clinical characteristic	n(%)	Blood glucose		χ2	P
		Normal	High		
Age					
<61 (y)ª	45(40.91)	16	29	0.267	0.599
≥61 (y)	65(59.09)	20	45		
Gender					
Male	68(61.82)	23	45	0.097	0.755
Female	42(38.18)	13	29		
Tumor size					
<3 (cm)	56(50.91)	26	30	9.727	**0.002**
≥3 (cm)	54(49.09)	10	44		
Tumor location					
Head	73(66.36)	24	49	0.002	0.963
Body and tail	37(33.64)	12	25		
Pathology type					
PDAC	102(92.73)	33	69	0.089	0.765
Other	8(7.27)	3	5		
Histological grade					
Poor	27(24.55)	9	18	0.006	0.938
Moderate/Well	83(74.45)	27	56		
TNM stage(UICC)					
I/II	62(56.37)	15	47	4.700	**0.030**
III/IV	48(43.63)	21	27		
Metastasis					
+	22(20.00)	3	19	4.552	**0.033**
-	88(80.00)	33	55		
Lymphatic metastasis					
+	19(17.27)	2	17	5.142	**0.023**
-	91(82.73)	34	57		
Perineural invasion					
+	22(20.00)	2	20	6.978	**0.008**
-	88(80.00)	34	54		

ª Median age

The bold values mean their P-values <0.05

### Cell lines and reagents

PC cell lines AsPc-1, BxPc-3, CFPAC-1, MiaPaCa-2, Panc-1 and SW1990 were obtained from Type Culture Collection of the Chinese Academy of Sciences (Shanghai, China) and cultured in appropriate media with 10% fetal bovine serum (FBS) (HyClone, Logan, UT, USA), 100 U/mL penicillin and 100 *μ*g/mL streptomycin (Gibco, Grand Island, NY, USA). Cells were cultured in medium containing increasing concentrations of glucose (5.5 mM to 25 mM) and added 19.5 mM mannitol to normal glucose medium as an osmotic pressure control. Rapamycin was purchased from HanBio Technology Co. Ltd. (HanBio, Shanghai, China), and mannitol, chloroquine and streptozotocin (STZ) were purchased from Sigma-Aldrich (St. Louis, MO, USA).

### Western blotting assay

Cells were cultured under specific conditions, and total protein was extracted using RIPA lysis buffer (Beyotime, Guangzhou, China). Then, western blotting assays were performed as previously described [30]. Primary antibodies against SREBP1, proliferation cell nuclear antigen (PCNA) and β-actin were purchased from Santa Cruz Biotechnology (Santa Cruz, CA, USA). Antibodies against Bcl-2, Bax, Beclin-1, p62 and LC3 were purchased from Abcam (Cambridge, UK) and those against Beclin-1 was purchased from Cell Signaling Technology (Danvers, MA, USA). Goat anti-rabbit and goat anti-mouse secondary antibodies were purchased from Cell Signaling Technology.

### Cell viability assay

Cells were plated in 96-well plates at a density of 5000 cells per well and treated with medium containing different concentrations of glucose for 24, 48 and 72 h. At the indicated times, we added methyl thiazolyl tetrazolium (MTT) to the cells and cultured them for an additional 4 h. Absorbance at 490 nm was detected using a multifunction microplate reader (POLARstar OPTIMA; BMG, Offenburg, Germany).

### Apoptosis assay

After culturing under the indicated conditions, cells were collected and stained with an Annexin V-FITC/7-AAD apoptosis detection kit (Becton Dickinson and Company, Franklin Lakes, NJ, USA). The percentage of apoptotic cells was detected by flow cytometry; Annexin V-FITC+/7-AAD- staining represented early apoptotic cells, and Annexin V-FITC+/7-AAD+ staining represented late apoptotic cells.

### Colony formation assay

Cells were diluted to the proper density, and samples of 1000 cells each were seeded in 35-mm Petri dishes. The medium was replaced with specific medium containing different concentrations of glucose. After culture for 2 weeks, the cells were fixed with 4% paraformaldehyde and stained with a 0.1% crystal violet solution. Images were captured with the ChemiDoc XRS imaging system (Bio-Rad Laboratories, Hercules, CA, USA).

### Immunofluorescence

Cells were cultured in different media for 6 h and fixed with 4% paraformaldehyde. Triton X-100 (0.3%) was used to increase membrane permeability, and the cells were blocked with 5% bovine serum albumin (BSA) at room temperature for 1 h. The cells were then incubated with primary antibodies at 4 °C overnight. The next day, the cells were incubated with secondary antibodies at room temperature for 1 h, and the nuclei were stained with DAPI for 15 min. A Zeiss Instruments confocal microscope was used for visualizeation.

### Autophagy detection by the mRFP-GFP adenoviral vector

Ad-mRFP-GFP-LC3 was purchased from HanBio Technology Co. Ltd. (HanBio, Shanghai, China). PC cells were transfected as previously described [32] and then cultured with different concentrations of glucose for 6 h. Images were taken with a Zeiss Instruments confocal microscope at 400× magnification.

### Transmission electron microscopy

After treatment, tumor cells were collected and fixed with 2.5% glutaraldehyde in 0.1 M cacodylate buffer for 2 h at room temperature. Next, 1% OsO4 was added and incubated for 1 h at room temperature. The samples were dehydrated with graded ethanol and embedded with low viscosity resin. Ultrathin stained sections were prepared and stained with 2% uranyl acetate and lead citrate for 30 min. Finally, autophagosomes were observed with a transmission electron microscope (H-7650, HITACHI, Tokyo, Japan) at 80 kV, and images were captured at a magnification of 40,000 × .

### Diabetes mouse model and subcutaneous transplantation model

BALb/c nude mice (male, 4 weeks old) were purchased and housed in the Animal Center of the Medical College of Xi’an Jiaotong University. All animal experiments were conducted according to the ethical guidelines established by the relevant Ethics Committee of the First Affiliated Hospital of Xi’an Jiaotong University. Mice were divided randomly into two groups, and fasting blood glucose (FBG) levels were detected after a 12-h fast. The mice in the high glucose group received an intraperitoneal injection of STZ (freshly prepared and dissolved in sodium citrate buffer) at a dose of 150 mg/kg; mice in the normal glucose group received an intraperitoneal injection of sodium citrate buffer alone. After 7 days, FBG levels were detected. The high glucose group and normal glucose group were then divided into two groups of 5 mice each.

After grouping, 1 × 10^6^ BxPc-3 cells with or without SREBP1 knockdown were implanted subcutaneously into the limbs of the mice. At the end of the experiment, all mice were euthanized. Tumors were collected for further study, and tumor weights were measured.

### Statistical analysis

Statistical analysis was performed using the SPSS software package (SPSS18.0; SPSS Inc., Chicago, IL, USA). Data are shown as the mean ± s.d., and Student’s t-test, one-way ANOVA followed by LSD post hoc test, the Kaplan-Meier method and Chi-square tests were applied. *P* < 0.05 was considered statistically significant. Each experiment was performed at least three times.

## Results

### Relationship between blood glucose level and the clinicopathologic characteristics of PC patients

**Fig. 1:**
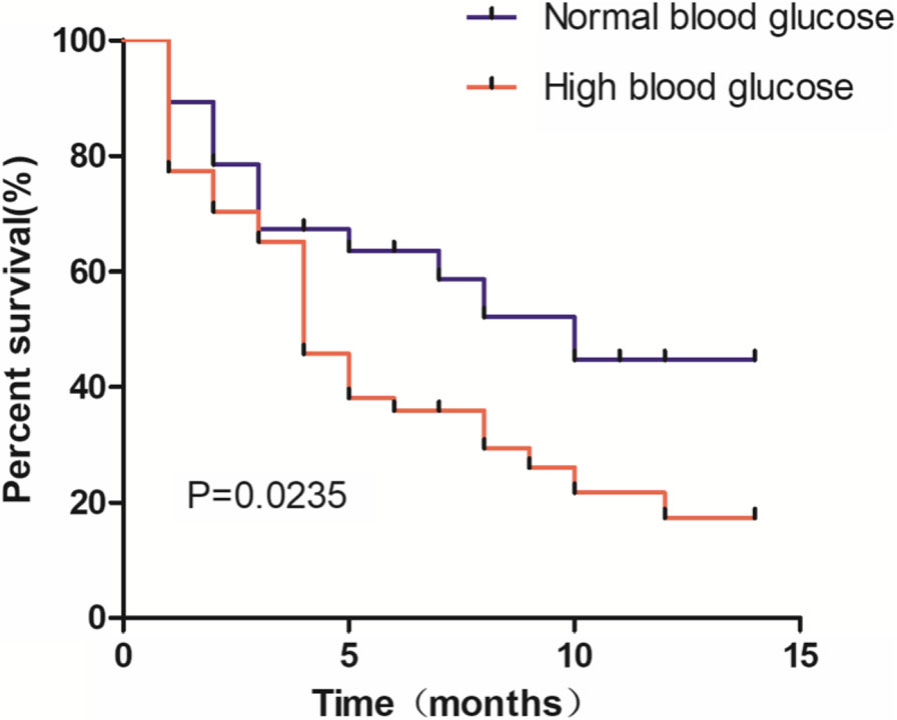
Prognostic significance of blood glucose levels in PC patients. Patients with high blood glucose levels showed a worse prognosis than patients with normal blood glucose levels

We collected clinical data for 110 patients and divided patients into two groups based on glucose level. And we analyzed the relationship between blood glucose level and clinical pathological features. As shown in Table 1, blood glucose levels were correlated with tumor size, TNM stage, metastasis, lymphatic metastasis and perineural invasion. However, no correlation with age, gender, tumor size, tumor location, pathology type, or histological grade were found. In addition, we followed all 110 patients from Jan 2016 to Mar 2017 then analyzed and plotted survival curves. As shown in Fig. 1, the total survival time was longer for the normal blood glucose group than for the high blood glucose group (*P* = 0.0235); the median survival time was 10 months for the former but only 4 months for the latter. These results show that blood glucose levels are closely related to the clinical pathological features of PC patients and have an effect on patient survival time.

### SREBP1 is overexpressed in PC tissues and cell lines

**Fig. 2:**
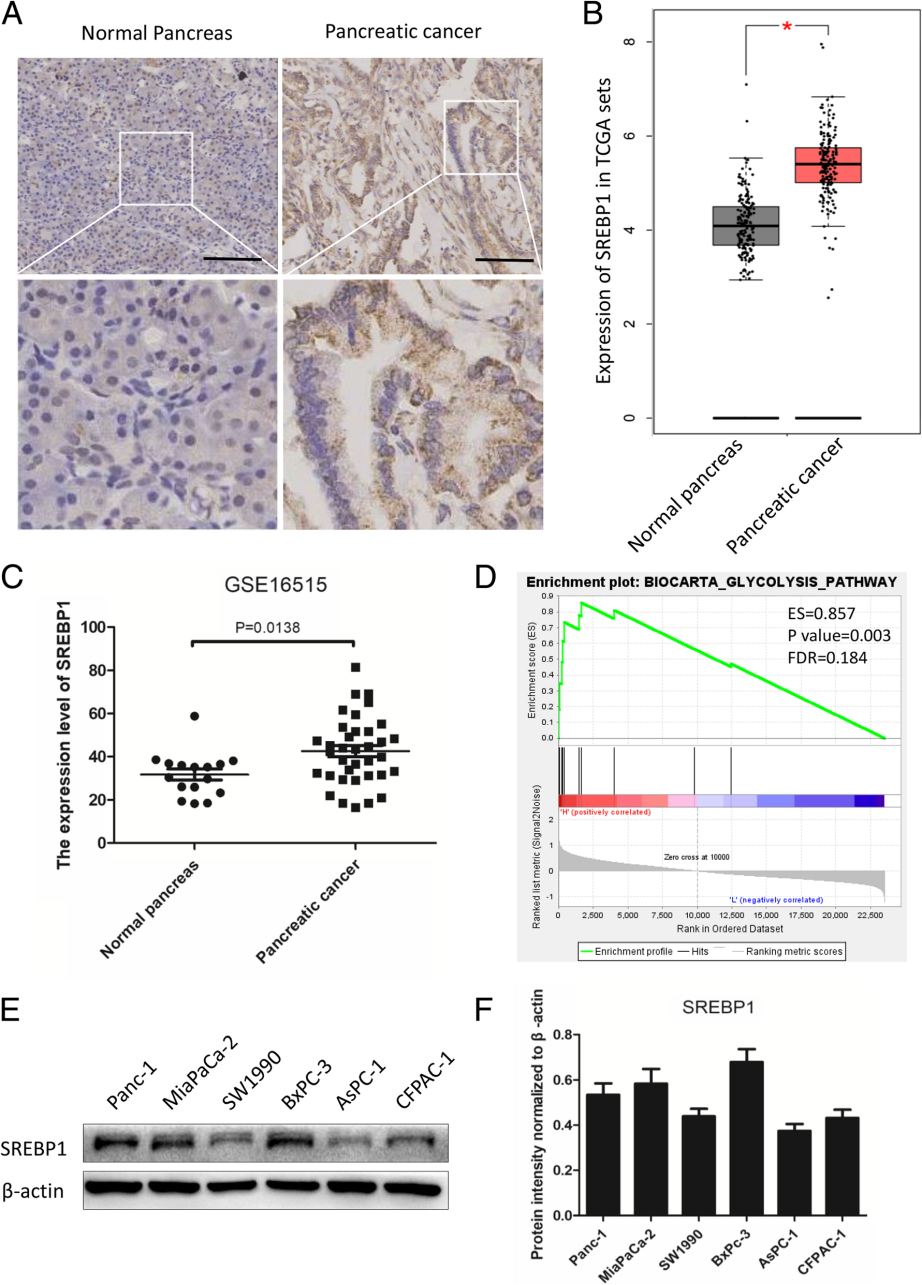
SREBP1 is overexpressed in PC tissues and cell lines. **a** Representative IHC images for SREBP1 in normal pancreas tissues and PC tissues. Scale bars: 100 μm. **b** The expression level of SREBP1 was detected in TCGA and GTEx. **c** The expression level of SREBP1 was detected in the GEO database (GSE16515, *P* = 0.0138). **d** Enrichment plots of GSEA correlation analyses for SREBP1 with glycolysis using the GSE16515 dataset (ES = 0.857, *P* = 0.003, FDR = 0.184). **e**,**f** Expression level of SREBP1 in six PC cell lines. β-actin was used as an internal loading control. *P < 0.05

Although SREBP1 has been found to play a crucial role in the progression of tumors, less is known about its role in PC. In this study, we detected the expression level of SREBP1 in normal pancreas and PC tissues. As shown in Fig. 2a, the expression level of SREBP1 was higher in PC tissue than in normal pancreas tissue. To confirm our results, we detected the expression level of SREBP1 using data from the Cancer Genome Atlas (TCGA) and GTEx, (normal pancreas, *n* = 171, and PC, *n* = 179). According to the results, SREBP1 was upregulated in PC tissue compared to normal pancreas tissue (*P* < 0.05, Fig. 2b). We also analyzed data from the GEO database (GSE16515, normal pancreas, *n* = 16 and PC, *n* = 36), and found that the expression level of SREBP1 in normal pancreas and PC tissues was 31.63 ± 10.19 and 42.50 ± 15.57, respectively (*P* = 0.0138, Fig. 2c). Next, we performed gene set enrichment analysis (GSEA) showing that SREBP1 expression was related to glycolysis (ES = 0.857, *P* value = 0.003, FDR = 0.184, Fig. 2d). Subsequently, we detected the expression level of SREBP1 in six PC cell lines (Fig. 2e and f). Specifically, a comparatively higher level of SREBP1 was observed in BxPc-3, MiaPaCa-2 and Panc-1 cells than in SW1900, AsPC-1 and CFPAC-1 cells. Thus, we chose BxPc-3 and MiaPaCa-2 cells for subsequent experiments. In summary, SREBP1 is overexpressed in both PC tissues and PC cell lines.

### High glucose promotes proliferation and suppresses apoptosis in PC cells

**Fig. 3:**
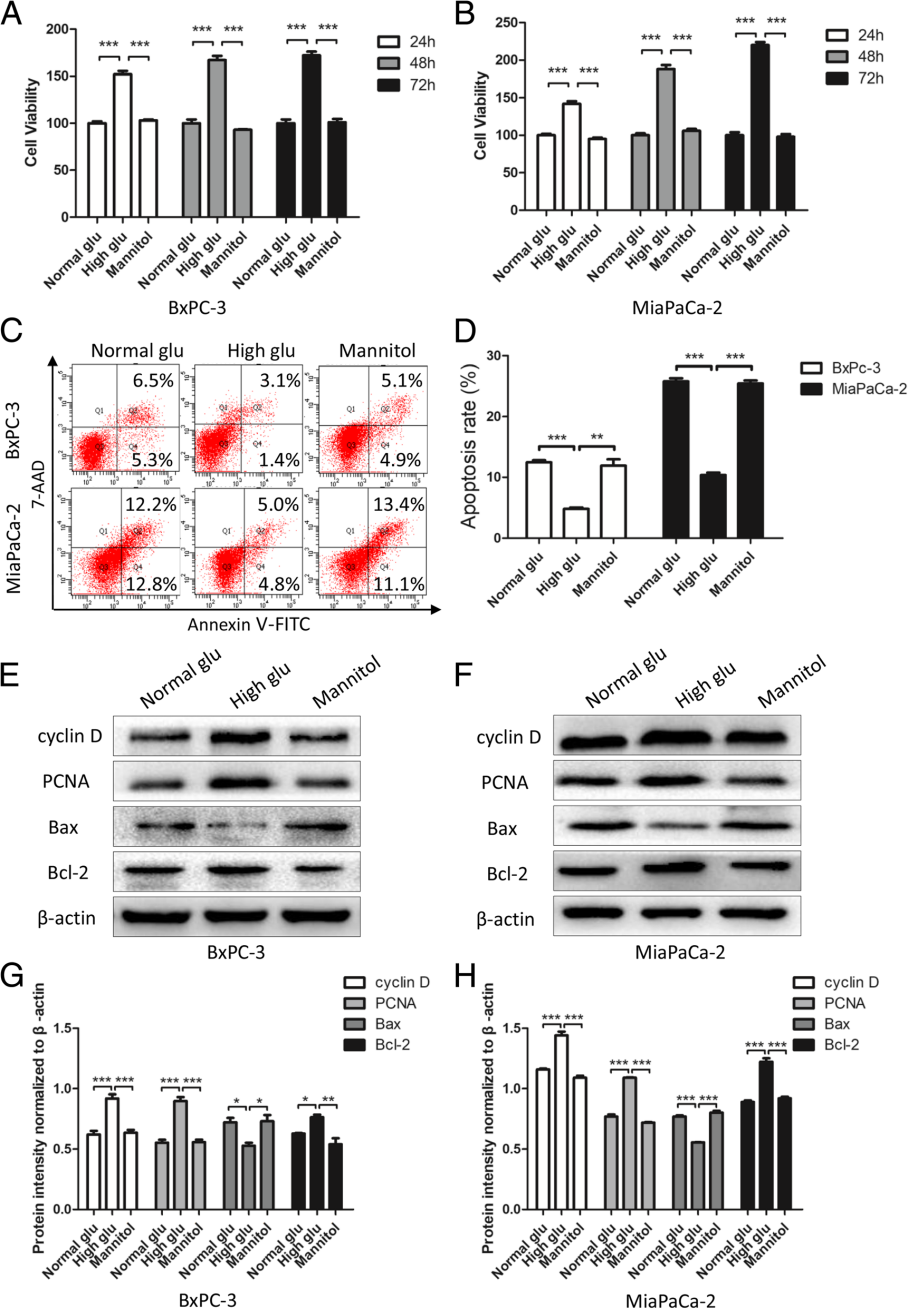
High glucose promotes proliferation and suppresses apoptosis in PC cells. **a**, **b** PC cells were treated with specific glucose levels and mannitol. At the indicated time points (24 h, 48 h and 72 h), cell viability was detected by MTT assays. **c**, **d** Effects of normal glucose, high glucose and mannitol on apoptosis in BxPc-3 and MiaPaCa-2 cells were detected by flow cytometry. **e**-**h** PC cells were treated with normal glucose, high glucose and mannitol for 48 h, and western blotting assays were performed to detect the expression of cyclin D, PCNA, Bax and Bcl-2; β-actin was used as an internal loading control. n = three independent experiments, *P < 0.05, **P < 0.01, or ***P < 0.001

To imitate both normal and high blood glucose microenvironments, we cultured PC cells in medium with specific concentrations of glucose. To exclude the osmotic pressure effect of high glucose, we added mannitol to the normal glucose medium as an osmotic control. We performed MTT assays to examine the relationship between high glucose and the proliferation of PC cells. We found that when treated with high glucose, the PC cell lines BxPc-2 and MiaPaCa-2 showed a much higher proliferation rate than when cultured in medium with normal glucose and mannitol (*P* < 0.001), with no difference between normal glucose and mannitol (Fig. 3a and b); these results are consistent with those of our previous study [33]. Additionally, we evaluated the apoptosis rate of PC cells under different glucose levels. As shown in Fig. 3c and d, the apoptosis rate was lower under high glucose than under normal glucose and mannitol, though there was no difference between normal glucose and mannitol, thus indicating that high glucose can suppress apoptosis. We also measured the expression level of proliferation and apoptosis markers, and as expected, high glucose promoted the expression of cyclin D, PCNA, and Bcl-2 and suppressed that of Bax compared to normal glucose; mannitol had no effect on proliferation or apoptosis (*P* < 0.05, Fig. 3e-h). These results confirm that high levels of glucose promote proliferation and suppress apoptosis in PC cells.

### High glucose increases SREBP1 expression and decreases autophagy

**Fig. 4:**
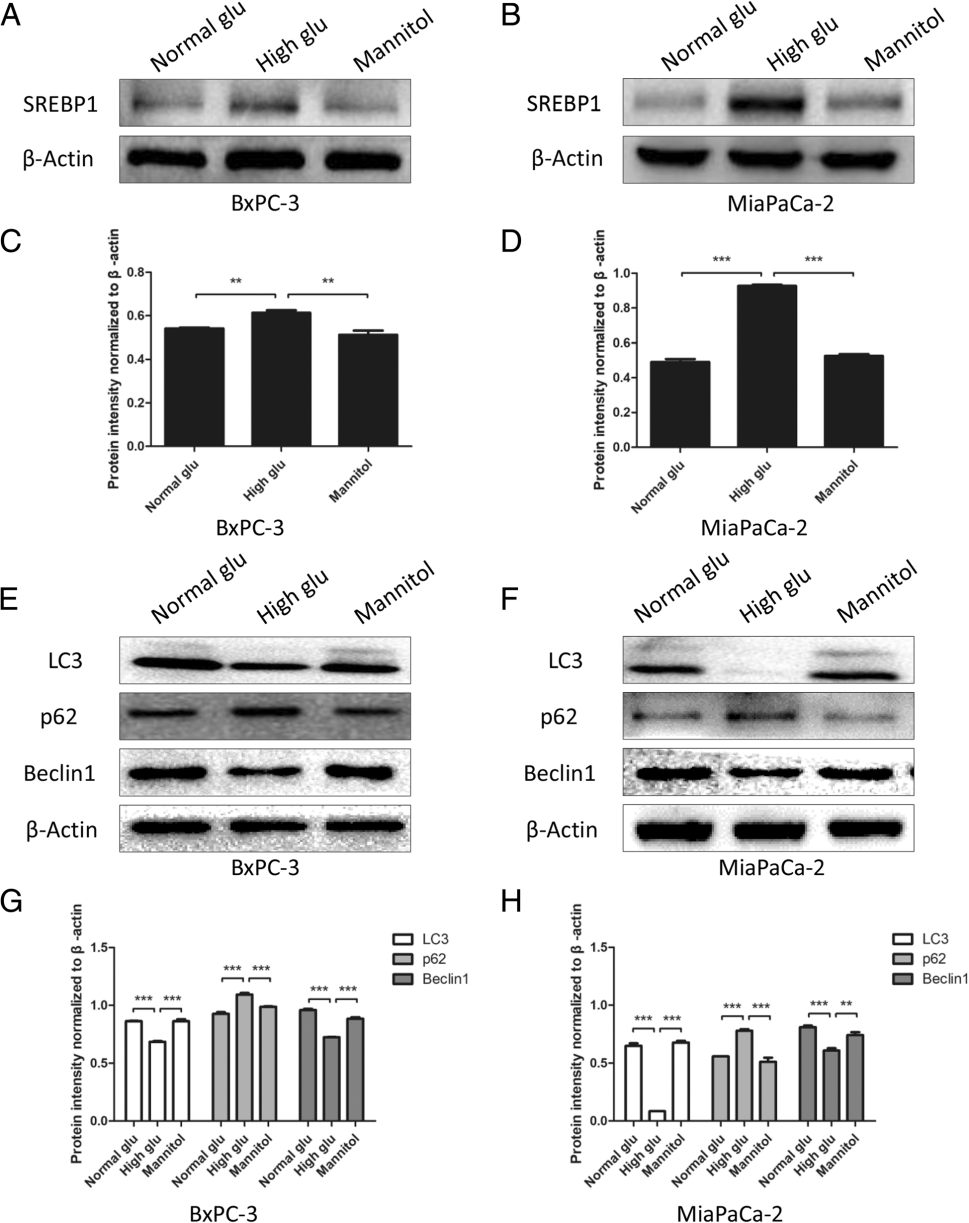
High glucose increases SREBP1 expression and decreases autophagy. **a**-**d** The expression of SREBP1 was detected by western blotting assays after PC cells were cultured with normal glucose, high glucose and mannitol. **e**-**h** PC cells were treated with normal glucose, high glucose and mannitol for 6 h, and western blotting assays were performed to detect the expression of LC3, p62 and Beclin1; β-actin was used as an internal loading control. n = three independent experiments, **P < 0.01, or ***P < 0.001

To investigate whether glucose has an effect on SREBP1 expression, we performed western blotting assays. Interestingly, we found that under high glucose, the expression of SREBP1 was expressed in greater abundance than when under normal glucose; mannitol had no effect on SREBP1 expression (*P* < 0.01, Fig. 4a-d). Furthermore, we performed western blotting to detect autophagy markers, such as LC3, Beclin-1 and p62. The results showed that the autophagy level was suppressed under high glucose compared to normal glucose, and the effect of osmotic pressure was excluded (P < 0.01, Fig. 4e-h). Taken together, these data show that high glucose plays an important role in regulating SREBP1 expression and autophagy level.

### High glucose promotes proliferation and suppresses apoptosis by upregulating SREBP1

**Fig. 5:**
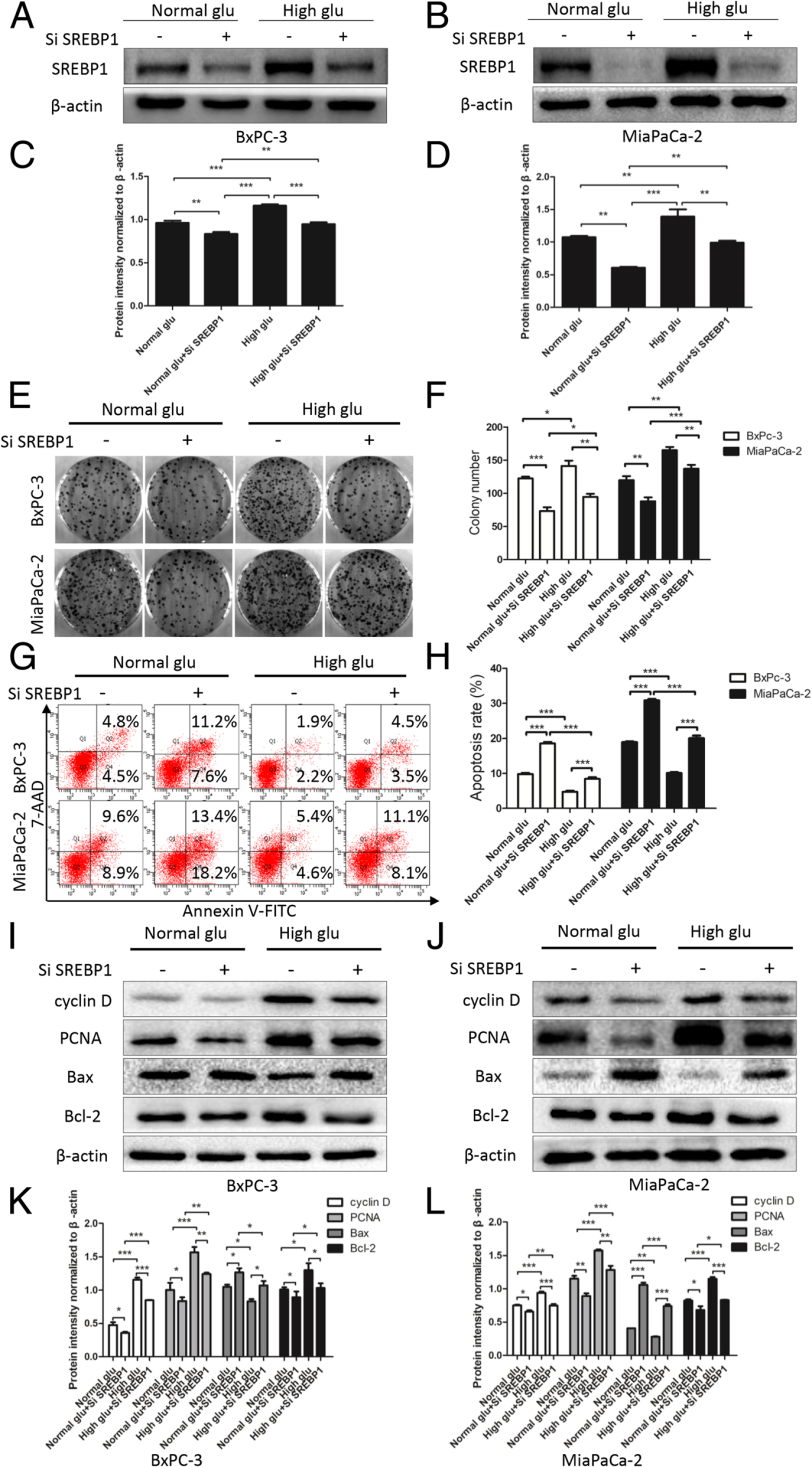
High glucose promotes proliferation and suppresses apoptosis by upregulating SREBP1. **a**-**d** After pretreatment with SiSREBP1 and culture with normal glucose and high glucose, the SREBP1 expression was detected by western blotting assays. Colony formation assays (**e**, **f**) and flow cytometry (**g**, **h**) were used to evaluate the effect of SiSREBP1 on colony-forming ability and apoptosis. (**i**-**l**) The expression of proliferation and apoptosis markers was detected by western blotting assays after pretreated with SiSREBP1 and culture under normal glucose and high glucose for 48 h. n = three independent experiments, *P < 0.05, **P < 0.01, or ***P < 0.001

Although our results indicate that the glucose supply is of vital importance to the processes of proliferation and apoptosis, it is unclear whether it functions through SREBP1 regulation. To address this, we used siRNA in our next experiment. As shown in Fig. 5a-d, SREBP1 expression was suppressed by siRNA under both normal glucose and high glucose conditions (*P* < 0.01). Furthermore, knocking down SREBP1 reversed the promoting effect on clone formation ability induced by high glucose (*P* < 0.05, Fig. 5e and f). We also detected the apoptosis rate of PC cells and found that knocking down SREBP1 enhanced apoptosis under both normal glucose and high glucose (*P* < 0.001, Fig. 5g and h). Similarly, western blotting assays confirmed that knocking down SREBP1 reduced the expression level of cyclin D, PCNA, and Bcl-2 and increased that of Bax, indicating that knocking down SREBP1 had a reversible effect on the proliferation promotion and apoptosis inhibition induced by high glucose (P < 0.05, Fig. 5i-l). We conclude that high glucose enhances proliferation and suppresses apoptosis in PC tumor cells by regulating SREBP1.

### High glucose regulates autophagy by upregulating SREBP1 expression

**Fig. 6:**
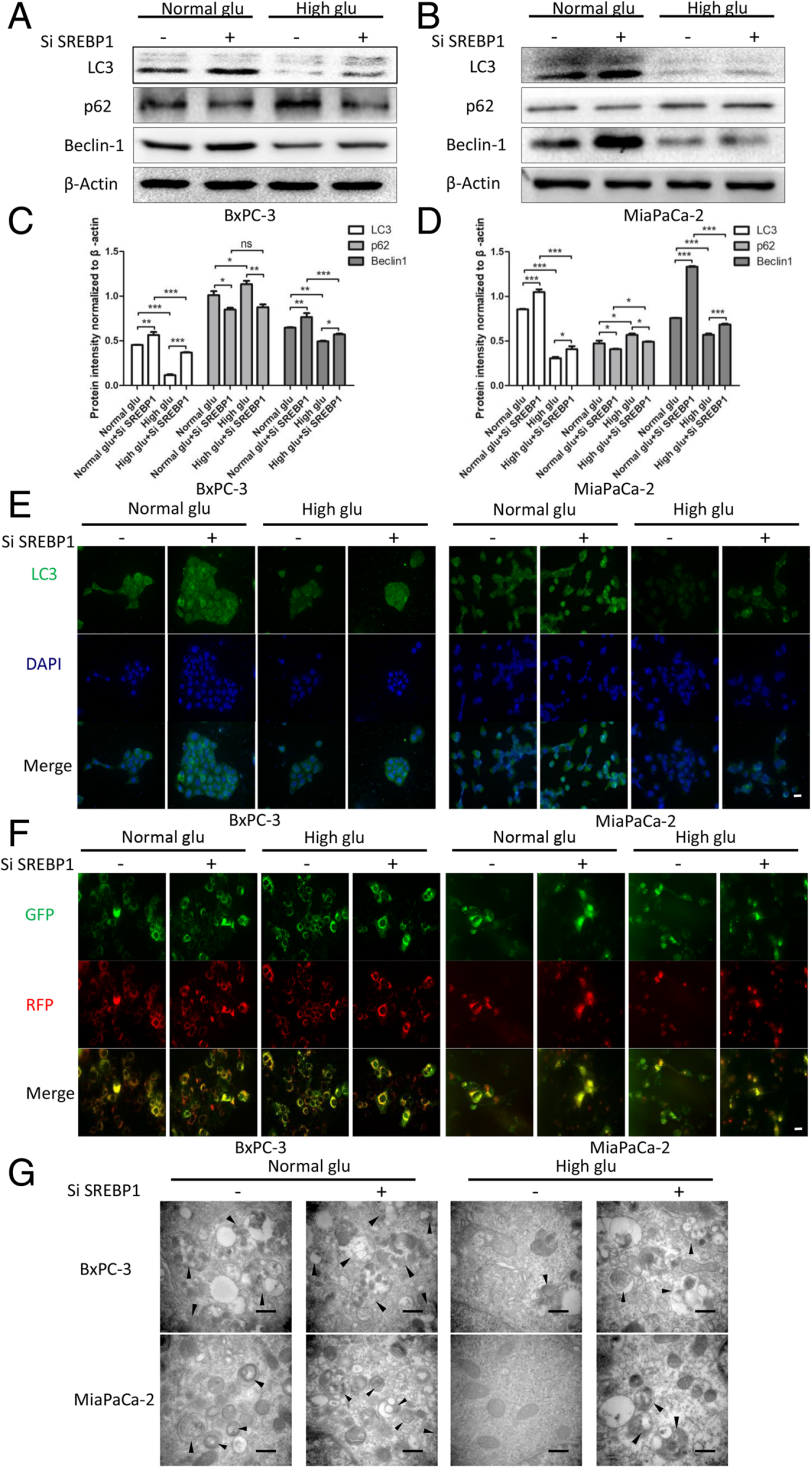
High glucose regulates autophagy by upregulating SREBP1 expression. **a**-**d** After knocking down SREBP1 and culture with normal glucose and high glucose medium, the expression level of autophagy markers was detected by western blotting assays; (**e**) LC3 expression was detected by immunofluorescence, green represents LC3, and blue represents nuclei, scale bars = 20 μm; (**f**) Autophagic flux was detected via diploid adenovirus (mRFP-GFP-LC3); red dots represent autolysosomes and yellow dots autophagosomes, scale bars = 20 μm; and (**e**) Autophagosomes were detected by electron microscopy (black arrow shows autophagosome), scale bars = 500 nm. n = three independent experiments, ns, not significant, *P < 0.05, **P < 0.01, or ***P < 0.001

Our results indicated that high glucose is able to regulate SREBP1 expression and autophagy, though it had not yet been elucidated whether high glucose controls the level of autophagy by regulating SREBP1. After knocking down SREBP1, the level of autophagy was increased under both normal glucose and high glucose (Fig. 6a-d). Immunofluorescence assays were then performed to detect the expression level of LC3, whereby green represents LC3 foci and blue nuclei, and we obtained the same results as with western blotting. Specifically, knocking down SREBP1 suppressed LC3 expression under both high glucose and normal glucose (Fig. 6e). Because the process of autophagy is dynamic, we used a diploid adenovirus (mRFP-GFP-LC3) to indicate autophagic flux: red dots represent autolysosomes and yellow dots autophagosomes. The results indicated suppression of autophagic flux under high glucose but knocking down SREBP1 reversed this effect induced by glucose (Fig. 6f). We next employed electron microscopy analysis because this technique is thought to be the gold standard for detecting autophagosomes. The results showed that there were fewer autophagosomes under conditions of high glucose compared to normal glucose. In addition, suppressing SREBP1 expression increased the number of autophagosomes (Fig. 6g). These data demonstrate that high glucose regulates autophagy by upregulating SREBP1.

### Activation of autophagy induces SREBP1 expression and suppresses apoptosis

**Fig. 7:**
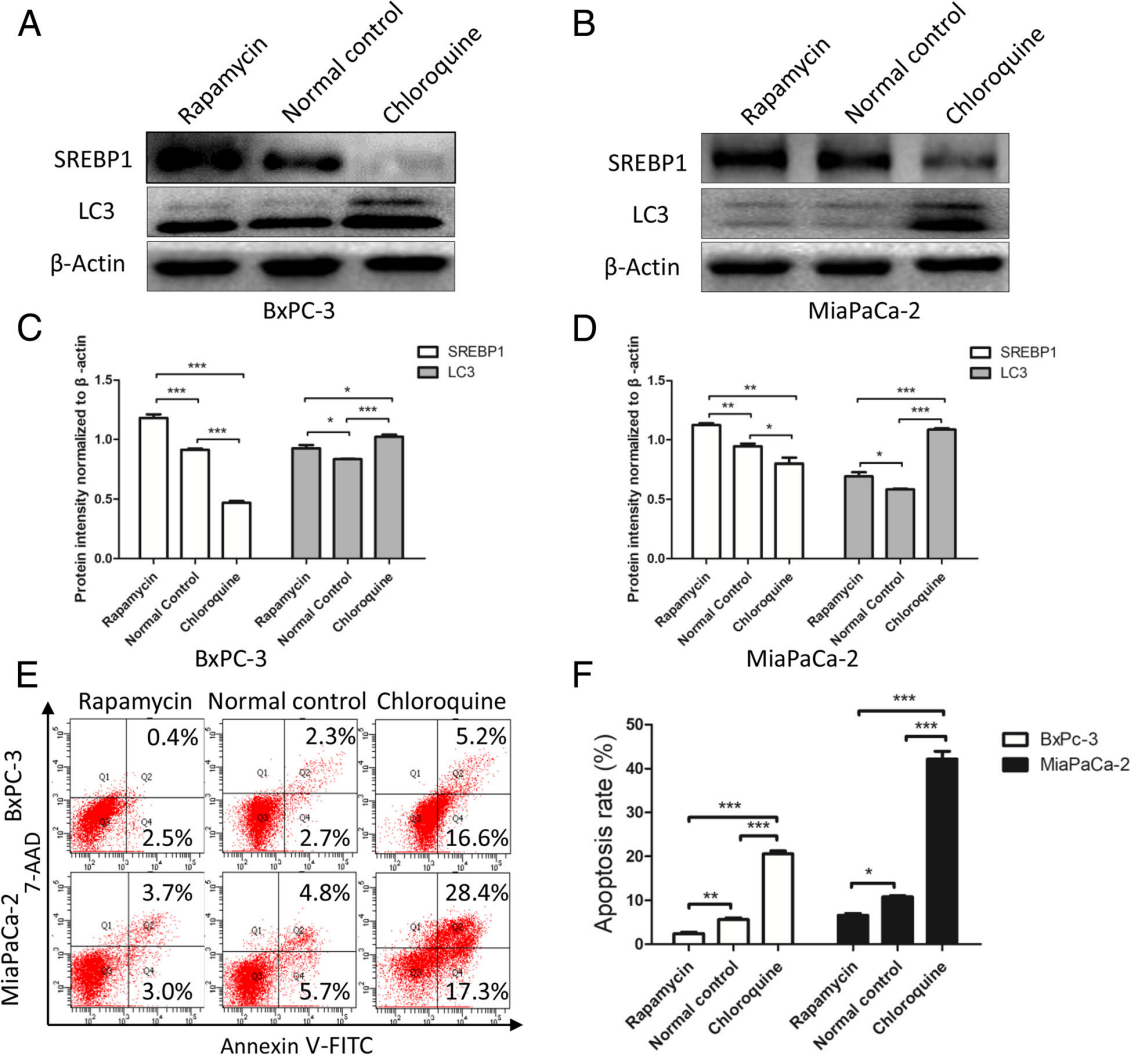
Activation of autophagy induces SREBP1 expression and suppresses apoptosis. **a**-**d** After altering the level of autophagy, SREBP1 expression and autophagy were detected by western blotting assays. **e**, **f** Apoptosis was detected by flow cytometry after autophagy levels were altered. n = three independent experiments, *P < 0.05, **P < 0.01, or ***P < 0.001

The results showed that the level of SREPB1 expression is inversely related to autophagy, yet, it remains unclear how autophagy influences SREBP1 expression. Thus, we used rapamycin (an autophagy agonist) and chloroquine (an autophagy inhibitor) to modulate autophagy levels. Western blotting assays were performed to detect SREBP1 expression, and as shown in Fig. 7a-d, the expression level of SREBP1 increased as the level of autophagy increased. At the same time, when autophagy was inhibited, SREBP1 expression was decreased. We also detected apoptosis in PC cell lines and found that activation of autophagy suppressed apoptosis but that suppression of autophagy promoted apoptosis (Fig. 7e and f). All of the above observations strongly suggest that increased autophagy induces SREBP1 expression and suppresses apoptosis.

### High glucose promotes tumor growth in vivo via SREBP1

**Fig. 8:**
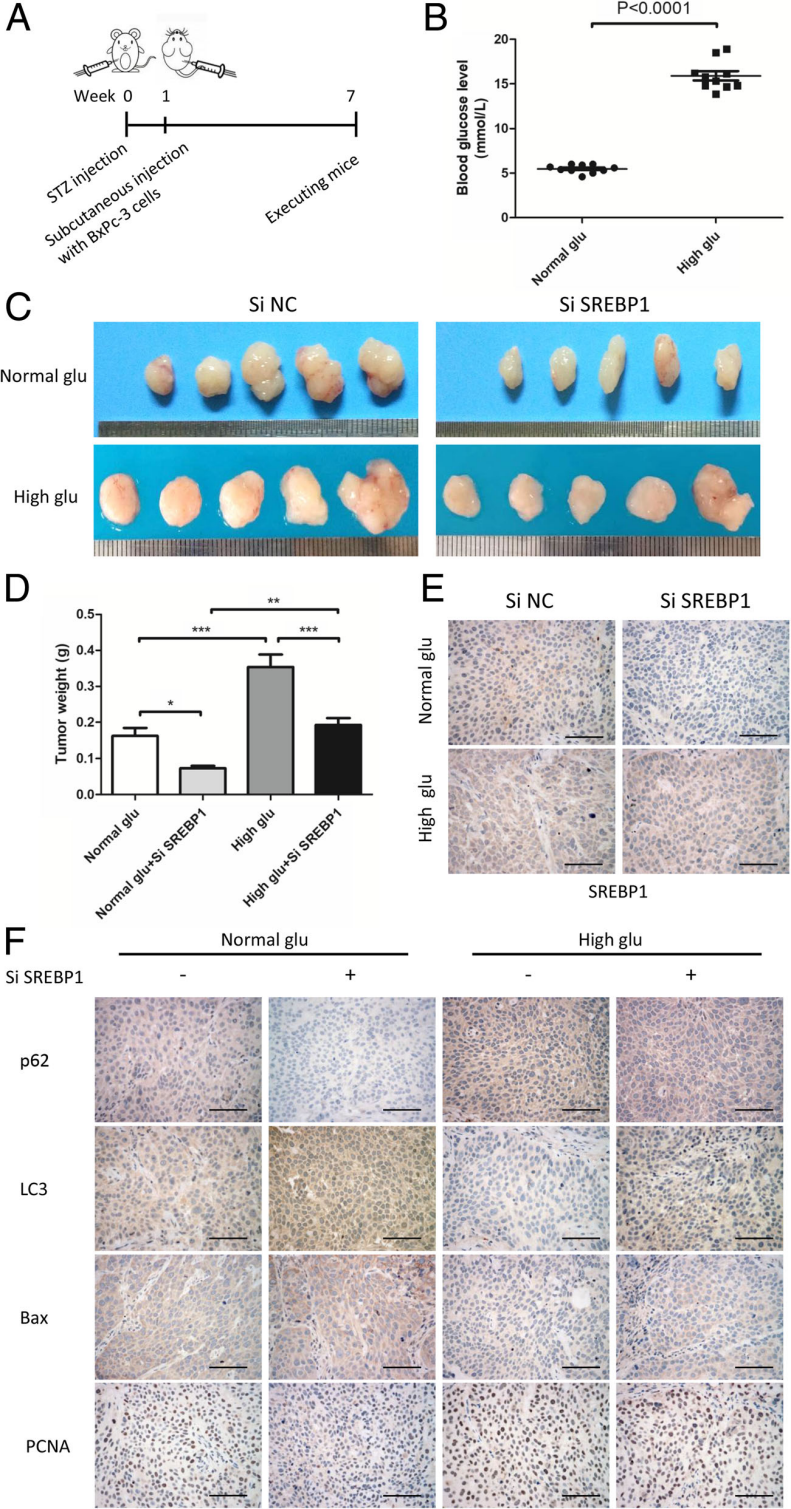
High glucose promotes tumor growth in vivo via SREBP1. **a** Schematic presentation of the induction of type 2 diabetes in mice and establishment of subcutaneous transplantation tumors. **b** Blood glucose levels of mice in the normal glucose group and high glucose group. **c** Macroscopic pictures of tumors from the groups normal glucose + Si NC, normal glucose + Si SREBP1, high glucose + Si NC, and high glucose + Si SREBP1 (*n* = 5 each group). **d** The weights of tumors in different groups. **e** SREBP1 expression was detected by IHC. **f** P62, LC3, Bax and PCNA expression was detected by IHC. Scale bars = 100 μm. n = three independent experiments, *P < 0.05, **P < 0.01, or ***P < 0.001

Based on our in vitro results, we performed an in vivo study to explore the role of high glucose and its mechanism. A schematic presentation of the induction of type 2 diabetes in mice and the establishment of subcutaneous transplantation tumors is shown in Fig. 8a. First, we established a type 2 diabetes mouse model by STZ injection (150 mg/kg) and measured the blood glucose levels on the 7th day, (*P* < 0.0001; Fig. 8b). We then established a subcutaneous transplantation tumor model with BxPc-3 cells with or without SREBP1 knockdown. At 6 weeks after tumor cell injection, the mice were euthanized, and we collected the tumor samples from all groups. As shown in Fig. 8c, tumors in the high glucose group were larger than those in the normal glucose group. Furthermore, tumors in the Si SREBP1 group were smaller than those in the Si NC group, regardless of the glucose conditions. Next, we measured tumor weights, and the results were in accordance with the macroscopic images (Fig. 8d). Immunohistochemical staining was also performed to detect the expression levels of SREBP1 and autophagy, proliferation and apoptosis markers. As expected, we found SREBP1, p62 and PCNA to be upregulated in the high blood glucose group and LC3 and Bax to be downregulated (Fig. 8e and f). In addition, knocking down SREBP1 increased the expression of LC3 and Bax while decreasing that of p62 and PCNA (Fig. 8e and f). These data demonstrate that high glucose promotes tumor growth in vivo by upregulating SREBP1 expression and suppressing autophagy.

## Discussion

**Fig. 9:**
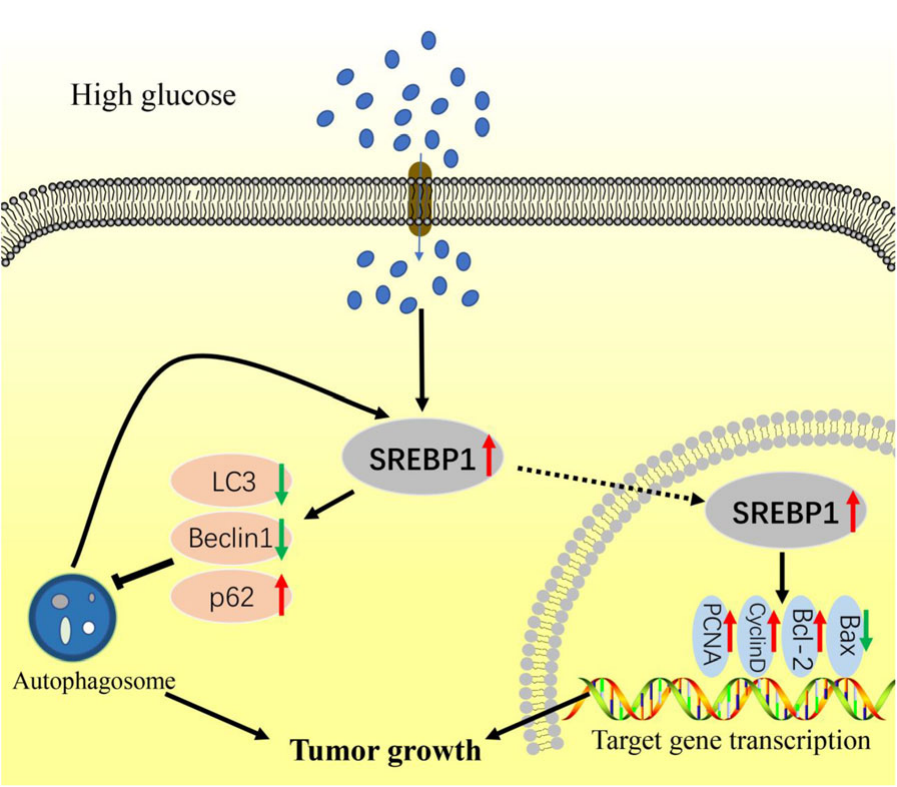
Schematic diagram illustrating how high glucose promotes PC growth. In high glucose microenvironment, SREPB1 is stimulated, after which proliferation is induced and apoptosis and autophagy are suppressed. Furthermore, autophagy promotes SREBP1 expression. Ultimately, this signaling cascade promotes PC growth

PC is a highly malignant cancer with high mortality [1, 3]. Several risk factors have been identified, including diabetes, but the specific mechanism remains unclear. SREBP1, a transcription factor in lipid metabolism, has been found to be important in the malignant progression of many types of tumors [26, 34–38], though little is known about how SREBP1 functions in PC. In this study, we found that high levels of blood glucose are closely related to a poor prognosis in PC patients. Furthermore, high glucose microenvironment upregulates SREBP1 expression and suppresses autophagy, thereby accelerating PC progression (Fig. 9).

Accumulating evidence has shown that diabetes provides a suitable microenvironment during the initiation and progression of PC [39]. Based on our previous study, high glucose conditions promote proliferation [33, 40], EMT [13], invasive and migratory activities [12] and perineural invasion [15]. Nonetheless, there is still a need for further investigation. In the present study, we confirmed the effect of high blood glucose on PC patient prognosis and that high glucose microenvironment accelerates proliferation and inhibits apoptosis. In addition, we explored the possible mechanism by which high glucose regulates proliferation and apoptosis, and we found that high glucose microenvironment causes autophagy.

According to existing research, the role of autophagy in cancer varies under different conditions [41, 42]. Due to the break down of damaged organelles and maintenance of homeostasis involved, autophagy is thought to have a protective role. However, autophagy also helps prevent cancer cells from being killed when they are starved or treated with anti-cancer drugs, which ultimately leads to cancer progression. Moreover, autophagy levels differ among cell types in high glucose microenvironment. Specifically, in podocytes, autophagy is induced by high glucose conditions and plays a protective role in podocyte injury [43]. Conversely, a lack of glucose supply induces autophagy in the breast cancer cell line MCF-7, protecting the cells from starvation [44]. In light of our findings, autophagy appears to be suppressed in PC under high glucose conditions but not under normal glucose conditions. This is mainly because the high glucose microenvironment provides cancer cells with adequate energy, enabling tumor growth [45]. In general, it remains to be determined which stage of autophagy is most influenced.

As a key transcription factor in lipid metabolism, SREBP1 has been thoroughly studied, yet it still needs to be elucidated how it functions in cancer. SREBP1 is a cancer-promoting gene in colorectal cancer, prostate cancer, breast cancer, endometrial carcinoma and nasopharyngeal carcinoma [28, 34, 35, 38]. Previously, we demonstrated that by suppressing PC stemness, SREBP1 acts as an effective chemotherapy sensitizer [30]. In the present study, we demonstrated that high glucose conditions promote proliferation and suppress apoptosis by upregulating SREBP1. In addition, SREBP1 mediates autophagy in PC cells. Specifically, higher levels of SREBP1 expression lead to lower levels of autophagy. Moreover, inhibiting or increasing autophagy results in changes in SREBP1. In summary, these results illustrate the existence of a negative feedback relationship between SREBP1 and autophagy.

Furthermore, we confirmed our findings in vivo using a diabetes mouse model. We found that high glucose microenvironment promotes tumor growth and that knocking down SREBP1 reversed this promoting effect of high glucose. According to IHC results, high glucose suppressed autophagy levels in vivo, and this effect was reversed by knocking down SREBP1. These findings reveal that SREBP1 is of vital importance in the process of high glucose-induced autophagy suppression in vivo.

## Conclusion

Overall, our observations demonstrate that high blood glucose levels are associated with poor prognosis in PC patients. High glucose conditions promote proliferation and suppress autophagy by increasing the expression of SREBP1. Moreover, a negative feedback loop exists between SREBP1 and autophagy. This study provides new insight into how high glucose promotes tumor progression and suggests that SREBP1 is a promising target for PC prevention and treatment.

## Data Availability

All data generated or analyzed during this study are available from the corresponding author on reasonable request.

## Abbreviations

BSA: Bovine serum albumin
DM: Diabetes mellitus
EMT: Epithelial-mesenchymal transition
FBG: Fasting blood glucose
FBS: Fetal bovine serum
GSEA: Gene set enrichment analysis
MTT: Methyl thiazolyl tetrazolium
PC: Pancreatic cancer
PCNA: Proliferation cell nuclear antigen
ROS: Reactive oxygen species
SREBP1: Sterol regulatory element-binding protein 1
STZ: Streptozotocin
TCGA: The Cancer Genome Atlas
